# Ultrasensitive Strain Sensor Based on Pre-Generated Crack Networks Using Ag Nanoparticles/Single-Walled Carbon Nanotube (SWCNT) Hybrid Fillers and a Polyester Woven Elastic Band

**DOI:** 10.3390/s21072531

**Published:** 2021-04-04

**Authors:** Yelin Ko, Ji-seon Kim, Chi Cuong Vu, Jooyong Kim

**Affiliations:** 1 Department of Organic Materials and Fiber Engineering, Soongsil University, Seoul 06978, Korea; yelinko@ssu.ac.kr (Y.K.); vuchicuong@soongsil.ac.kr (C.C.V.); 2 Department of Smart Wearables Engineering, Soongsil University, Seoul 06978, Korea; js23kim@soongsil.ac.kr

**Keywords:** strain sensor, elastic band, woven fabric, pre-crack generation, single-walled carbon nanotubes (SWCNTs), silver pastes, e-textile

## Abstract

Flexible strain sensors are receiving a great deal of interest owing to their prospective applications in monitoring various human activities. Among various efforts to enhance the sensitivity of strain sensors, pre-crack generation has been well explored for elastic polymers but rarely on textile substrates. Herein, a highly sensitive textile-based strain sensor was fabricated via a dip-coat-stretch approach: a polyester woven elastic band was dipped into ink containing single-walled carbon nanotubes coated with silver paste and pre-stretched to generate prebuilt cracks on the surface. Our sensor demonstrated outstanding sensitivity (a gauge factor of up to 3550 within a strain range of 1.5–5%), high stability and durability, and low hysteresis. The high performance of this sensor is attributable to the excellent elasticity and woven structure of the fabric substrate, effectively generating and propagating the prebuilt cracks. The strain sensor integrated into firefighting gloves detected detailed finger angles and cyclic finger motions, demonstrating its capability for subtle human motion monitoring. It is also noteworthy that this novel strategy is a very quick, straightforward, and scalable method of fabricating strain sensors, which is extremely beneficial for practical applications.

## 1. Introduction

Wearable electronics have attracted tremendous attention for their potential applications in effective interactions between humans and smart systems, including personal healthcare, human–machine interfaces, and electronic skins [1,2,3,4,5,6,7,8]. Among these, flexible and stretchable strain sensors have seen a rapid rise in demand because of their roles in monitoring physical activities, as well as health-related variables, such as body motions [1], human expressions [2], breathing [5], and pulse [6]. Various types of novel strain sensors have emerged based on changes in their electrical characteristics in response to mechanical deformations, which can be described as resistive [1,6], capacitive [7], or piezoelectric [8] effects. In particular, resistive-type strain sensors are of great interest owing to their facile fabrication process and simple readout systems.

As the change in resistance under strain depends upon the evolution of conductive networks within the sensors [9], the structural design of sensing materials to impart conductivity, as well as substrate flexibility, is an important consideration. Multiple nanomaterials, including carbon nanotubes (CNTs) [5,9,10], graphene [11,12,13], nanowires [2], and conductive polymeric materials [14] have been widely explored for generating conductive pathways. Among those, CNTs have been recognized as one of the most promising nanomaterials for highly stretchable strain sensors because of their outstanding mechanical and electrical properties, which are due to generation of a highly interconnected percolation network [15]. However, it has been noted that strain sensors developed from these carbon nanomaterials suffer from low sensitivity. On the contrary, silver nanoparticles have demonstrated their exceptionally high sensitivity when used as conductive fillers in strain sensors, while exhibiting poor stretchability [16].

Flexible polymers, such as Ecoflex [10], polydimethylsiloxane (PDMS) [2], thermoplastic polyurethane (TPU) [9], and textiles [17], are popular substrates for strain sensors. Here, the simplicity of the methodologies for embedding active nanomaterials into flexible substrates is a crucial determinant of the possibility for scaling-up production and practical applications. Along with other approaches, dip-coating is a widespread and economical technique for depositing target materials onto substrates; however, it also causes relatively weak bonding [18]; thus, it may not be effective in developing high-performance sensors.

High sensitivity is one of the most desirable features of a strain sensor because it offers opportunities to explore subtle strains; hence, significant efforts have been made in this regard. For example, Li et al. [19] showed that structuring micro-sized pores into CNT/PDMS nanocomposites can significantly enhance sensitivity—from 3.2 to 15—by reducing the conductive pathways. Shi et al. [20] introduced fullerene as a lubricant agent into a nanocomposite comprising silver nanowires and graphene oxide to facilitate slippage of the films. This increased the sensitivity of the resulting strain sensor to 2392.9. The mechanical fragmentation process was also demonstrated to increase the sensitivity of the graphene-foam-based strain sensor, from 2.2 to 29, owing to the enlarged contact area between the adjacent components [13]. However, these attempts require complicated or time-consuming processes and the synthesis of complex materials.

Pre-crack generation is another technique for increasing the sensitivity of a strain sensor by intentionally creating defects on its surface during manufacturing processes; the corresponding sensing mechanism relates to the disconnection/reconnection of crack junctions during the tensile process [21]. The prebuilt cracks are formed by laser engraving, followed by roll-to-roll pressing [22,23], bending on a curvature surface [24], or pre-stretching [6,21,25,26]. Among these, pre-stretching is significant because it utilizes a universal testing machine (UTM). As this machine is typically used to evaluate the performance of a resistive-type strain sensor, it does not require additional instruments for the sensitivity enhancement step. Previous research has verified the effectiveness of this strategy, reporting that pre-stretching can realize a high sensitivity of 87 [26], 1344.1 [25], and 83,982.8 [6].

Despite the great achievements in improving the sensitivity of strain sensors by the formation of prebuilt cracks, such a strategy with fabric substrates is a neglected research area. However, textile substrates offer not only simple fabrication processes but also substantial engineering possibilities at various levels of the structure hierarchy, ranging from fibers to yarns and then fabrics [27]. In terms of pre-crack generation, diverse interlocking networks within textile materials can provide a wide range of research opportunities, which inevitably affect the dynamics of crack creation, opening, and closing, determining the performance of a strain sensor. Finally, the exceptional properties of textiles, including wearability, conformability, deformability, and comfort, should be highlighted.

Herein, we present a facile method for the fabrication and sensitivity enhancement of a textile-based strain sensor: dip-coat-stretch strategy. Our sensor comprises a brittle and highly conductive layer (silver) on a stretchable and moderately conductive layer (single-walled CNT/polyester woven elastic band). The polyester woven elastic band utilized as the substrate of our sensor comprises rubber and polyester yarn. This textile is highly flexible, elastic, durable, and resilient. In addition, this band is lightweight, inexpensive, readily available on the market, and can be easily sewn to other fabrics, making it a promising substrate for electronic textiles. The pre-cracked strain sensor exhibited an ultra-high sensitivity of up to 3550 (1.5 to 5% strain), which addresses the issue of low gauge factor observed within small working ranges [19,28]. To expand the possible applications of our strain sensor to occupational settings, we also integrated it into firefighting gloves. The developed prototype detected finger motion, suggesting its application in smart protective gear, whose worth has been of less interest but deserves more attention, as highlighted by Chen and Lawo [29].

## 2. Materials and Methods

A commercially available polyester woven elastic band (PEB) was used as the substrate for the strain sensor in the present study. This fabric band is highly elastic, durable, wrinkleless, and resilient. The structure of PEB is a plain weave (polyester 80% and rubber 20%) consisting of polyester yarns as the warp and polyester-yarn-wrapped rubbers as the weft. The as-received PEB was cut into a rectangle of 1 cm × 4 cm using a laser cutting machine. The bands were rinsed several times with distilled water to remove the surface contaminants and were completely dried.

**Figure 1:**
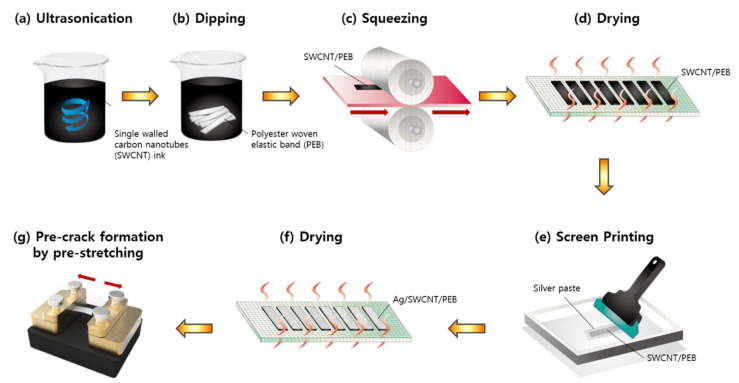
The manufacturing process for strain sensors based on polyester woven elastic band (PEB): (**a**) ultrasonication of single-walled carbon nanotube (SWCNT) ink; (**b**) dipping PEB into SWCNT ink; (**c**) squeezing SWCNT/PEB; (**d**) drying SWCNT/PEB; (**e**) screen printing for silver-paste coating; (**f**) drying Ag/SWCNT/PEB; (**g**) pre-crack formation by pre-stretching to make pre-cracked Ag/SWCNT/PEB.

The manufacturing process of the pre-cracked strain sensor is shown in Figure 1. The 0.1 wt % single-walled carbon nanotube (SWCNT) ink (KH Chemicals, Bundang, Korea) was ultrasonicated before use in a sonicator bath for 2 h at a temperature of 60 °C (Figure 1a). The band was dipped into the SWCNT inks for 1 min (Figure 1b) and squeezed by a roller to remove excess water at a speed of 1.0 m·min^−1^ and a cylinder pressure of 0.3 MPa (Figure 1c). This procedure was repeated four times to ensure that the SWCNT particles could penetrate and effectively attach to the PEB fibers. After SWCNT-dipped PEB (SWCNT/PEB) was two-way dried for 10 min at a temperature of 100 °C (Figure 1d), its upper side was coated with stretchable silver paste twice (DM-SIP-2001, Dycotec Materials, United Kingdom) via screen printing (Figure 1e). We considered that coating the samples with silver paste for one time or more than two times would be less effective because the resulting silver layer was too thin or too brittle for crack generation. Silver-coated SWCNT/PEB (Ag/SWCNT/PEB) was obtained using the same drying machine at the temperature of 120 °C for 15 min (Figure 1f).

To enhance the sensitivity of the sensor, we pre-stretched Ag/SWCNT/PEB to form prebuilt crack networks, with a loading and releasing rate of 80 mm·s^−1^, using a customized UTM (Dacell Co., Seoul, Korea) (Figure 1g). The pre-stretching speed in the current study was much faster than that adopted in other studies of 5 mm·min^−1^ [6] and 2 mm·min^−1^ [25]. This is attributed to the highly flexible and elastic characteristics of PEB, which make the sensitivity enhancement procedure much more facile and efficient. Finally, a pre-cracked strain sensor was prepared after being positioned in air at room temperature for 24 h to eliminate any possible internal stresses caused by the rapid extension. Five samples were obtained and the averaged data from these samples were used to assess the strain sensing performance.

## 3. Results and Discussion

### 3.1. Surface Structure of the Pre-Cracked Strain Sensor

**Figure 2:**
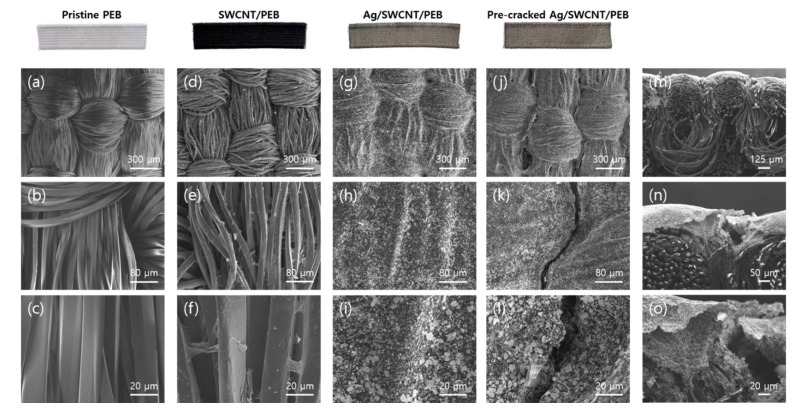
Top-view SEM images of pristine PEB at 300 um (**a**); 80 um (**b**); 20 um (**c**); SWCNT/PEB at 300 um (**d**); 80 um (**e**); 20 um (**f**); Ag/SWCNT/PEB at 300 um (**g**); 80 um (**h**); 20 um (**i**); Pre-cracked Ag/SWCNT/PEB at 300 um (**j**); 80 um (**k**); 20 um (**l**); Cross-sectional SEM images of pre-cracked Ag/SWCNT/PEB at 125 um (**m**); 50 um (**n**); 20 um (**o**).

The surface morphology of the pre-cracked strain sensor obtained by scanning electron microscopy (SEM) (JSM-7800F, JEOL Ltd., Tokyo, Japan) is shown in Figure 2. Pristine PEB had a typical woven structure (Figure 2a), and SWCNT particles were appended to the PEB fibers after the dipping and squeezing processes, forming a conductive network (Figure 2d–f), which was not observable in the pristine PEB (Figure 2b,c). We could observe that these processes did not alter the woven surface structure of the fabric substrate (Figure 2d). Silver paste covered the entire surface of the SWCNT/PEB during the screen printing process (Figure 2h,i) while also retaining the innate woven structure (Figure 2g). The pre-crack structure was distinctly present in the pre-cracked Ag/SWCNT/PEB mainly along the warp direction (Figure 2j,k), isolating the silver segments (Figure 2l). In the cross-sectional image, the bilayer structure of the prepared strain sensor was clearly observed with a silver layer of 100 um and SWCNT-embedded PEB of 1 mm (Figure 2m). While the flexible PEB fibers were adjacent to each other under no strain, the brittle silver layer was found to be broken along the warp direction (Figure 2n,o).

### 3.2. Sensitivity Enhancement by Pre-Crack Generation

**Figure 3:**
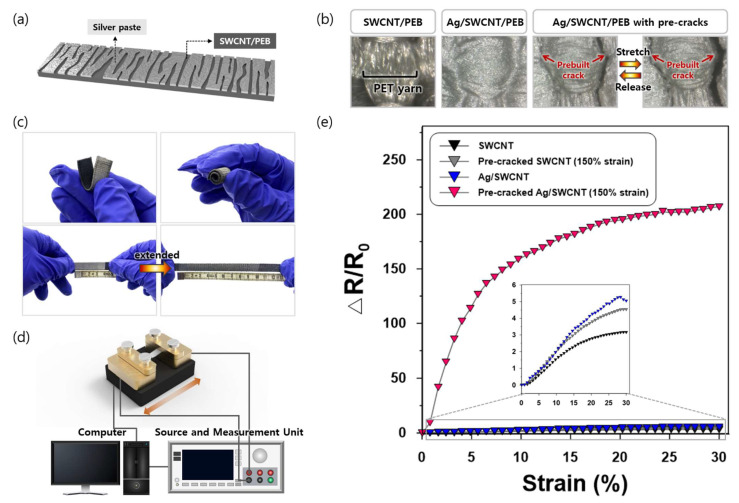
(**a**) Schematic structure of the pre-cracked strain sensor; (**b**) Pre-crack morphology of the pre-cracked strain sensor; (**c**) Flexibility of the pre-cracked strain sensor; (**d**) Customized Universal Testing Machine (UTM); (**e**) Relative changes in resistance versus strain of non-cracked SWCNT, pre-cracked SWCNT, non-cracked Ag/SWCNT, and pre-cracked Ag/SWCNT sensors.

Figure 3a shows a schematic representation of the pre-cracked strain sensor, which has a composite structure of PEB dipped with SWCNTs and a silver top layer. The cracks generated by pre-stretching were enlarged during the elongation process and were recovered when the sensor was released (Figure 3b). As shown in Figure 3c, our sensor was very flexible and stretchable up to 250%. In addition, SWCNT particles did not merely adhere to the fabric surface but penetrated into the PEB (Figure 3c) creating conductive networks inside the sensor, which can be distinguished from other works that sprayed [6] or deposited [23] active materials on their substrates. The strain sensing performance of the sensor was evaluated by the same UTM used for the pre-crack generation and a Keysight B2902A precision source and measurement unit (Figure 3d). Relative resistance changes were calculated by ΔR/R_0_, where R_0_ is the initial resistance of the sensor under no strain and ΔR is the absolute change in resistance when stretched.

The pre-cracked Ag/SWCNT sensor pre-stretched at 150% strain exhibited much greater sensing performance than the non-cracked and pre-cracked SWCNT sensors, and non-cracked Ag/SWNCT sensors (Figure 3e). With the prebuilt cracks on the silver layer, relative resistance change increased by approximately 50 times. This was much greater than the effects reported by other studies that also leveraged shape deformations to enhance the sensitivity: the introduction of micro-sized pores and mechanical fragmentation increased the sensitivity by approximately five times [17] and 15 times [13], respectively. Pre-crack generation improved the relative resistance changes of both Ag/SWCNT and SWCNT sensors; however, the enhancement effect was only notably significant when the pre-cracks were formed on the surface of the silver pastes. This can be explained by the brittle characteristics of the silver layer, rendering the sensor surface more beneficial for generating a large number of cracks along the woven PEB structure. In addition, the non-cracked Ag/SWCNT sensor showed a slight decrease in ΔR/R_0_ after an applied strain of 27–28% (Figure 3e). This could be due to the high elasticity of the PEB and the stiffness of the silver pastes, causing the samples to slip from the clamps of the UTM. In contrast, the pre-cracked Ag/SWCNT sensor displayed a steady increase in the same working range. This indicates that the creation of prebuilt cracks on a brittle layer with a textile substrate may be an effective strategy to enhance the stretchability of a strain sensor, as reported by Zhou et al. [6], who also adopted pre-stretching on the TPU substrate. Since our study focused on small strains, however, this possibility should be demonstrated by further investigations.

**Figure 4:**
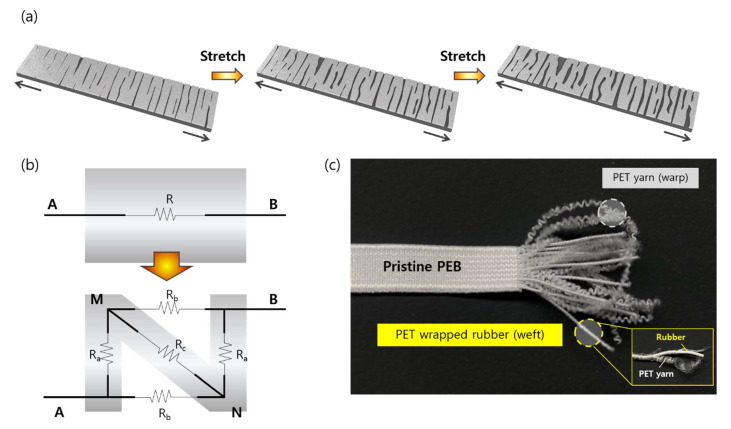
(**a**) Strain sensing mechanism of the pre-cracked strain sensor: the dark gray layer represents PEB dipped into SWCNT inks; the light gray layer shows the silver coating on SWCNT/PEB; (**b**) Resistance model of the crack structure; (**c**) Fabric structure of the polyester woven elastic band utilized in the present study.

The working principle of the pre-cracked strain sensor is illustrated in Figure 4a, which largely depends on the dimensional changes in the crack structures along the sensor’s surface. Under mechanical straining, a strain sensor senses resistance variations resulting from changes in conductive pathways; this is ascribed to electron tunneling, which emerges between adjacent conductive components [6]. These conductive pathways are further damaged as cracks are formed under stretching, decreasing the contact between the silver and SWCNT coating. The electrical resistance subsequently increases during the extension process. When a sample is released, the gaps created due to the elongation become narrow, the conductive pathways are recovered as the islands are reconnected, and the resistance gradually decreases. The cracks can repeatedly open and close according to loading and unloading, making the strain sensor reversible.

The resistance of the sensor structure before and after the prebuilt cracks were formed can be calculated using Equations (1) and (2), respectively (Figure 4b):

(1) $$R_{AB\left(before\right)}=R_{Ag\left(before\right)}+\;R_{SWCNT\left(before\right)}$$

where *R*_Ag_ and *R*_SWCNT_ represent the resistance of Ag and SWCNT before the cracks were generated, respectively.

(2) $$\begin{array}{c} R_{AB\left(after\right)}=f\left(R_{a},\;R_{b},\;R_{c}\right)=R_{A}+\frac{\left(R_{M}+R_{b}\right)\left(R_{N}+R_{a}\right)}{R_{M}+R_{b}+R_{N}+R_{a}}\\ with\;R_{A}=\;\frac{R_{a}R_{b}}{R_{a}+R_{b}+R_{c}},\;\;R_{M}=\;\frac{R_{a}R_{c}}{R_{a}+R_{b}+R_{c}},\;\;R_{N}=\;\frac{R_{b}R_{c}}{R_{a}+R_{b}+R_{c}} \end{array}$$

where *R_a_* represents the resistance of Ag and SWCNT between AM and BN after the cracks were generated, *R_b_* represents the resistance of SWCNT between AN and MB after the cracks, and *R_c_* represents the resistance of Ag and SWCNT between MN after the cracks.

The rapid pre-stretching and releasing before a sample undergoes the first extension as a strain sensor further produces breakages of islands on the surfaces, dynamically changing the connection, disconnection, and reconnection of the crack structures. Here, the brittle layer (silver-paste coating) and stretchable layer (SWCNT/PEB) interact in such geometrical changes of the structural cracks. The silver-paste coating has much higher conductivity than SWCNT/PEB, endowing low initial resistance to the pre-cracked strain sensor. During the elongation process, the breakage of the brittle silver layer cause significant increase in resistance. In the meantime, the stretchable but moderately conductive bottom layer (SWCNT/PEB) bridges the gaps between the prebuilt cracks on the brittle top layer, maintaining the conductive networks of the sensor.

Since the prebuilt cracks by pre-stretching are generated on the surface, structures of textile substrates are highly likely to affect the crack formation. Compared to silicone elastomer counterparts that typically have smooth surfaces, fabrics have dynamic variations in their surfaces depending on weaving or knitting methodologies, characteristics of fibers and yarns, and surface treatments during the manufacturing process. The woven structure of the PEB substrate comprising inner rubber in the weft (Figure 4c) can be particularly beneficial for fabricating pre-cracked strain sensors. The highly stretchable nature of the rubber helps to widen the prebuilt and newly created cracks quickly and efficiently, increasing the sensitivity of the sensor compared to that without pre-generated cracks. In addition, as the rubber is highly elastic, the recovery of the initial crack geometry and conductive pathways can also take place effectively. This can result in high stability and reversibility under repeated stretch and release cycles.

Another advantage of the textile substrate is that it does not require pre- and post-treatments in the sensor fabrication process, which is often mandatory when utilizing other silicone elastomer substrates. For example, Wang et al. [30] proposed a printed crack-based strain sensor with PDMS and addressed that the adhesiveness to the printing device as well as hydrophobicity of PDMS make it inevitable to perform surface treatments beforehand. However, textile-based strain sensors can be fabricated utilizing the textiles as received and dipping or immersing them into solutions with active materials [17,25,27]. In addition, the woven PEB does not shrink in the direction perpendicular to the elongation stress, while TPU mat was reported to show such deformation [6]. This dimensional stability may contribute to producing consistent output signals, increasing the stability of the pre-cracked strain sensors’ performance.

**Figure 5:**
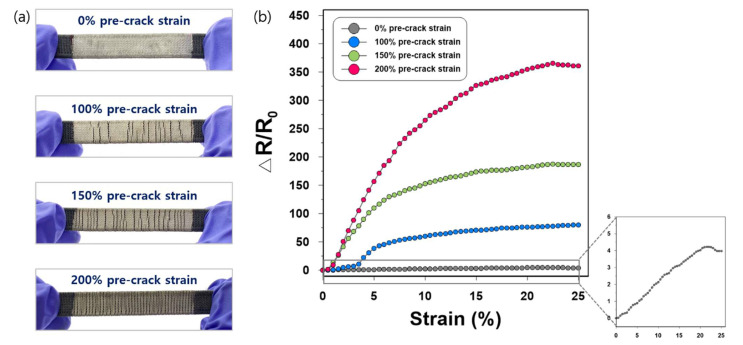
(**a**) Crack images for the pre-cracked strain sensors according to pre-stretching strains of 0%, 100%, 150%, and 200%; (**b**) Relative changes in resistance versus strain of samples pre-stretched at 0%, 100%, 150%, and 200% strain.

The relative changes in resistance under different pre-stretching strains (100%, 150%, and 200%) were investigated (Figure 5). We set the maximum pre-stretch strain at 200% because the samples were not firmly fixed by the clamps over that strain. As the pre-stretching strain increased, the prebuilt cracks formed more densely in the direction perpendicular to the stretch (Figure 5a). The prebuilt cracks did not necessarily appear at regular intervals on the PEB surface at strains of 100% and 150%. However, at 200% strain, the silver coating was broken along most of the warps, making the cracks more uniformly present. The uneven distribution of the prebuilt cracks formed by the 100% and 150% pre-crack strain should be noted as one of the limitations of the pre-stretching method, compared to another strategy to create prebuilt cracks such as the laser engraving method that can control the depth and interval of the cracks [22,23]. It would be worthwhile to further explore effective ways to enhance the controllability of the pre-stretching process.

As shown in Figure 5b, it is obvious that the pre-cracked strain sensor pre-stretched at 200% strain achieved a superior sensing performance (R_0_ of 10.6 Ω and ΔR of 3814.6) compared to other sensors with fewer cracks (pre-stretched at 150% strain: R_0_ of 10.4 Ω and ΔR of 1945.0; pre-stretched at 100% strain: R_0_ of 10.6 Ω and ΔR of 871.9), suggesting that more active opening of the prebuilt cracks is related to the increase in resistance. It is also notable that up to an applied strain of 4%, the crack formation by 100% pre-crack strain did not significantly enhance the performance of the sensor. This issue disappeared in the sensors with 150% and 200% pre-crack strain; the relative changes in resistance when stretched with small strains (within 5% applied strain) were the most rapid among other working ranges. This shows that our pre-crack formation on a rigid surface with sufficiently large pre-stretching strains is particularly effective for detecting subtle stretches.

The gauge factor (GF) is used to evaluate the sensitivity of strain sensors, which is defined as the ratio of the relative change in resistance to mechanical strain (GF = (ΔR/R_0_)/ε, where ε is the applied mechanical strain). The 200% pre-stretched strain sensor possesses a high sensitivity at an applied strain of 25%, with a GF of 1443. The gauge factors of the other pre-cracked sensors pre-stretched at 150%, 100%, and 0% strain were 747, 320, and 16, respectively. The ultra-high sensitivity of the pre-cracked Ag/SWCNT sensor is imputable to the high pre-stretching rate (maximum 200%) attained owing to the use of PEB as a flexible substrate. Previous studies that fabricated strain sensing composites by combining CNTs and other elastomers, such as TPU [6] and PDMS [26], adopted pre-stretching strains of 100% and 120%, respectively. In these studies, the CNT/TPU sensor had a GF of 428.5 at <100% applied strain [6] and the CNT films/PDMS sensor achieved a GF of 87 at <100% applied strain [26], which is lower than that of our sensor.

**Figure 6:**
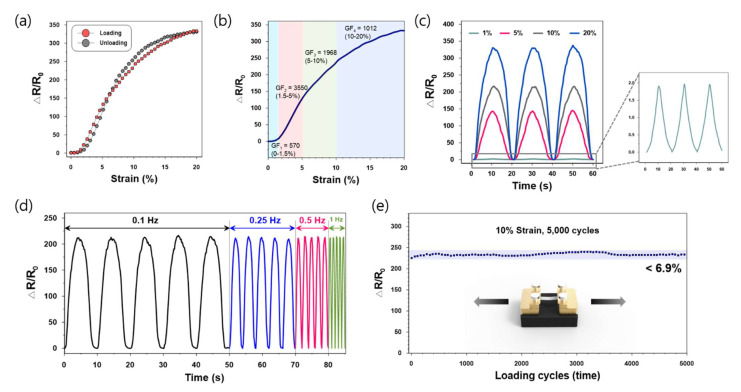
Strain sensing properties of the pre-cracked Ag/SWCNT sensor including (**a**) Hysteresis; (**b**) Gauge factors; (**c**) Signals at different loading strains; (**d**) Responses at different loading frequencies; (**e**) Durability after 5000 stretching–releasing cycles.

Figure 6c shows the electrical responses of the sensor under various dynamic strains, with a frequency of 0.01 Hz. The output signals were highly reversible and stable during the three stretching–releasing cycles, which could be due to the high elasticity of the sensor. In addition, our sensor exhibited clear signals even with the very small strains of 1%. To verify the stability of the sensor at higher frequencies, we also tested the dynamic responses by increasing the frequencies from 0.1 to 1 Hz (Figure 6d). The sensing patterns showed no obvious changes in amplitudes at various frequencies, demonstrating that the pre-cracked strain sensor can display stable performance over a broad frequency range. Dynamic durability indicates that a sensor can retain its performance under repetitive stretching–releasing cycles, which makes it appropriate for use in long-term applications. As shown in Figure 6e, the relative changes in the resistance of the sensor remained nearly unchanged, with less than 6.9% change after the 5000 loading cycles. (Figure 6e).

### 3.3. Woven Fabric Structure in Pre-Crack Generation

Commercial stretchable fabrics are typically manufactured in knitted structures with the integration of stretchable fibers or yarns, such as spandex, elastane, or natural rubbers. As the looping design of knitted fabrics makes them more stretchable than woven fabrics, stretchable knitted fabrics have been largely utilized as a platform material in previous studies on strain sensors. For example, Lee et al. [31] and Cai et al. [28] fabricated strain sensors by dipping a knitted fabric into a graphene oxide solution, with a subsequent reduction process. Vu and Kim [17] developed a strain sensor with knitted fabric as a flexible substrate and silver/SWCNT layers as active materials. The strain sensor proposed by Reddy et al. [27] was based on a commercial polyester elastic band similar to ours but the band also had a knitted structure.

**Figure 7:**
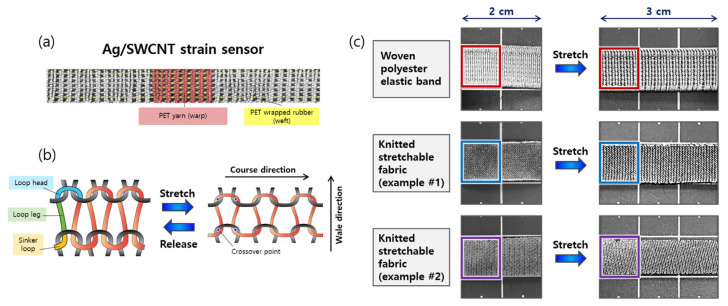
(**a**) Construction of the polyester woven elastic band utilized as the stretchable substrate; (**b**) Schematic diagram of an example knitted fabric in initial and stretched states; (**c**) Dimensional changes of polyester woven elastic band and knitted stretchable fabric examples under 50% applied strain.

However, given that a woven fabric contains elastic fibers, this structure may be particularly beneficial with regard to pre-crack generation on textile substrates. As shown in Figure 7a, two independent yarns comprise woven PEB in perpendicular directions: PET yarn (warp) and PET-wrapped rubber (weft). Under applied strain, the stretch and recovery of the rubber yarn are less dependent on other components (i.e., warp yarns). This helps the cracks in the brittle silver coating along the warp direction close and open much more easily. In contrast, a knitted fabric is based on interlocking loops (Figure 7b). The gaps between the loops can also increase when stretched; however, the extension around the loop head is limited because the adjacent sinker loop raises the friction at the crossover points [32], which may adversely affect crack propagation.

We simply compared the dimensional changes of our textile substrate (woven PEB) with two commercial stretchable fabrics (Figure 7c): knitted nylon fabric (nylon 85% and spandex 15%; example #1) and knitted polyester fabric (polyester 94% and spandex 6%; example #2). Each sample was pre-stretched to generate pre-cracks after being coated with silver paste. When stretched by 50% applied strain, the vertical unit of the woven PEB decreased by 31.6% (from 19 to 13 per 1 cm). The other two knitted stretchable fabrics had a decrease of 28.6% (from 21 to 15 per 1 cm, example #1) and 18.8% (from 16 to 13 per 1 cm, example #2), respectively.

This comparison indicates that our PEB substrate can be notably effective in widening cracks when stretched, decreasing conductive pathways and increasing the sensitivity of strain sensors. Additional highlights of a woven fabric compared to a knitted structure are that it is more durable, does not curl up when stretched, and can be more easily sewn on the clothes, which can contribute to enhancing the stability of the sensors. It should be noted that our current investigation (Figure 7c) is only preliminary; it does not consider diverse factors that would affect the propagation of prebuilt cracks, such as the proportion of elastic fibers, various weaving and knitting methodologies, size of yarns, and density of fabrics. The effects of structural differences of fabrics on the sensitivity enhancement by pre-crack generation should be further explored with systematic examinations, which will be the direction of our follow-up study.

While the woven structure and the inner rubber of PEB seem to significantly contribute to the sensitivity enhancement and high stability as well as reversibility of our pre-cracked strain sensor, its high elasticity caused the sensor to slip from the clamps during the performance testing. In other words, it was challenging to test the sensor beyond the applied strain of 30%, as the sensor exhibited its characteristics of quick recovery to the original state (i.e., not elongated state). It made the sensitivity enhancement by the prebuilt cracks to be tested in a limited working range in our sensor. Given the commonly known, high stretchability of commercial elastic band, we expect that pre-crack generation on a brittle layer that is positioned on a stretchable textile layer has the potential to achieve both high sensitivity and wider working ranges. Therefore, future investigations on pre-crack generation on woven PEB can explore the optimal point between its elasticity and stretchability, to develop a high-performance strain sensor with a wider working range. Diverse fabric properties, such as the ratio of elastane fibers, weaving method, and length as well as thickness of the substrate can be considered.

### 3.4. Application of the Pre-Cracked Strain Sensor

The advantages of our pre-cracked strain sensor can be summarized as follows: (1) it can be easily integrated into clothes as the sensor is based on flexible textiles; (2) it does not require a complicated manufacturing process or complex materials; and (3) it possesses ultra-high sensitivity in small strain ranges and high stability. Leveraging these points, we tested whether this strain sensor could be applied as a finger motion sensor when integrated into firefighting gloves. We did not directly mount the sensor on the skin as other studies did for subtle strain detection [26,27] because of the possibility that the conductive materials, especially SWCNTs, can be toxic to the skin. We considered that if our sensor could demonstrate itself to be sensitive enough to detect finger motions on bulky gloves, its possible motion sensing on thinner and fitting gloves (finger bending) or the skin (pulse, breathing, speaking, or swallowing) can also be indicated.

**Figure 8:**
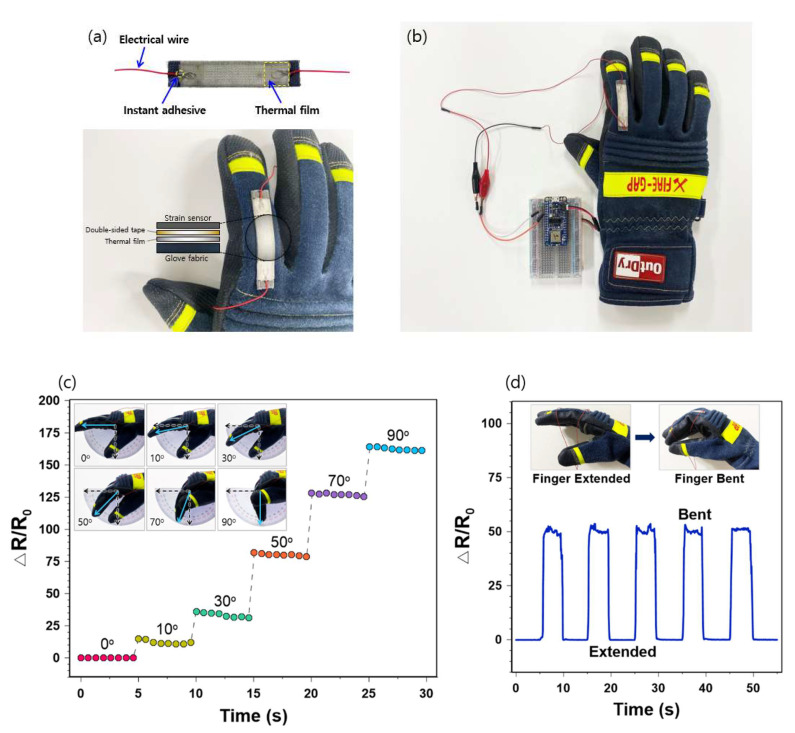
(**a**) Structure of the sensor integrated into the firefighters’ glove; (**b**) Prototype of the finger-motion-sensing firefighter’s glove; (**c**) Relative resistance changes of finger bending at different angles; (**d**) Relative resistance changes of cyclic finger motions of extending and bending.

To develop a portable prototype, electrical wires (AWG 32) were fixed on both edges of the sensor using an instant adhesive (Figure 8a). A thin thermal film (100% polyurethane; Sealon Ltd., Seoul, Korea) was subsequently attached to the sensor by a heat press (ISP-450MR, INNOSTA, Hanam, Korea) at 150 °C for 15 s to ensure a firm connection between the electrical wires and electrodes. When securing the sensor on the glove, a double-sided tape and an additional thermal film were placed on the fabric of the glove to enhance adhesion (Figure 8a). The prototype firefighting glove with the developed integrated sensor was prepared after connecting it to a hardware platform (Figure 8b). This platform consisted of an integrated microcontroller (MCU) with Bluetooth 5.0 (nRF52840), a reference resistor (~ 1 kΩ), and a lipo-battery (3.7 V). The MCU reads signals from the sensor and transmits them to a phone, desktop, or tablet monitor using a wireless protocol (Bluetooth low energy).

The finger movement detection of our sensor was tested on the firefighting glove (Figure 8c,d). Considering that grasping is one of the most typical tasks for protective gloves, we increased the finger bending angles from 0 to 90° (Figure 8c). The sensor signal immediately increased as the bending angle increased and the pre-cracked strain sensor was sensitive enough to detect subtle changes of finger angles during the test. At the end of each phase of the 5-s duration, the relative resistance changes slightly decreased. This can be explained by the rigid and bulky features of the protective glove, making it difficult to keep the finger bent in the glove over time.

The strain sensing performance during cyclic finger motion was also investigated (Figure 8d). As expected, the sensor exhibited clear patterns of increasing and decreasing resistance with bending and extension, without any apparent changes in the amplitude. The sensitive and repeatable finger-motion-detecting activities of our sensor suggest its promising applications to protective gloves where exact monitoring of finger movements in these gloves is needed. Whereas the signals were stable during the extending period, the resistance changes were relatively unstable during the bending period. Again, this can be attributable to the stiffness of the tested protective glove, making it challenging to maintain the same finger positions during the test. Therefore, possible noises should be considered and addressed when integrating highly sensitive strain sensors to protective gloves.

## 4. Conclusions

In conclusion, we proposed a pre-cracked textile-based strain sensor fabricated using a quick, easy, and straightforward strategy. By dipping, coating, and pre-stretching, the commercial fabric elastic band was fabricated into a strain sensor with excellent sensing performance. The sensor was comprised of two different layers: a brittle and highly conductive layer (silver coating) where the prebuilt cracks were formed, and a stretchable and moderately conductive layer (SWCNT/PEB) that bridged the gaps between those cracks under strain. Our sensor exhibited ultra-high sensitivity with a GF of 570 within 0–1.5%, 3550 for 1.5–5%, 1968 for 5–10%, and 1012 for 10–20% working ranges. In addition, the sensor possessed notable flexibility, low hysteresis, and high reversibility, stability, and durability owing to the highly stretchable and elastic nature of the substrate band. The sensor could also monitor finger bending movements, even when integrated into the rigid and bulky firefighting glove. This strain sensor’s facile, scalable, and quick fabrication process and excellent performance, as well as textile-based properties, will provide promising advantages for a wide range of applications, including the development of smart protective gears.

## Data Availability

Not applicable.
